# Cuprous Oxide Thin Films Implanted with Chromium Ions—Optical and Physical Properties Studies

**DOI:** 10.3390/ijms23158358

**Published:** 2022-07-28

**Authors:** Katarzyna Ungeheuer, Konstanty W. Marszalek, Marzena Mitura-Nowak, Piotr Jelen, Marcin Perzanowski, Marta Marszalek, Maciej Sitarz

**Affiliations:** 1 Faculty of Computer Science, Electronics and Telecommunications, AGH University of Science and Technology in Krakow, 30-059 Krakow, Poland; marszale@agh.edu.pl; 2 The Henryk Niewodniczanski Institute of Nuclear Physics Polish Academy of Sciences in Krakow, 30-236 Krakow, Poland; marzena.mitura-nowak@ifj.edu.pl (M.M.-N.); marcin.perzanowski@ifj.edu.pl (M.P.); marta.marszalek@ifj.edu.pl (M.M.); 3 Faculty of Materials Science and Ceramics, AGH University of Science and Technology in Krakow, 30-059 Krakow, Poland; pjelen@agh.edu.pl (P.J.); msitarz@agh.edu.pl (M.S.)

**Keywords:** ion implantation, copper oxide, thin films

## Abstract

Cuprous oxide is a semiconductor with potential for use in photocatalysis, sensors, and photovoltaics. We used ion implantation to modify the properties of Cu_2_O oxide. Thin films of Cu_2_O were deposited with magnetron sputtering and implanted with low-energy Cr ions of different dosages. The X-ray diffraction method was used to determine the structure and composition of deposited and implanted films. The optical properties of the material before and after implantation were studied using spectrophotometry and spectroscopic ellipsometry. The investigation of surface topography was performed with atomic force microscopy. The implantation had little influence on the atomic lattice constant of the oxide structure, and no clear dependence of microstrain or crystalline size on the dose of implantation was found. The appearance of phase change was observed, which could have been caused by the implantation. Ellipsometry measurements showed an increase in the total thickness of the sample with an increase in the amount of implanted Cr ions, which indicates the influence of implantation on the properties of the surface and subsurface region. The refractive index *n*, extinction coefficient *k*, and absorption coefficient optical parameters show different energy dependences related to implantation dose.

## 1. Introduction

**Figure 1:**
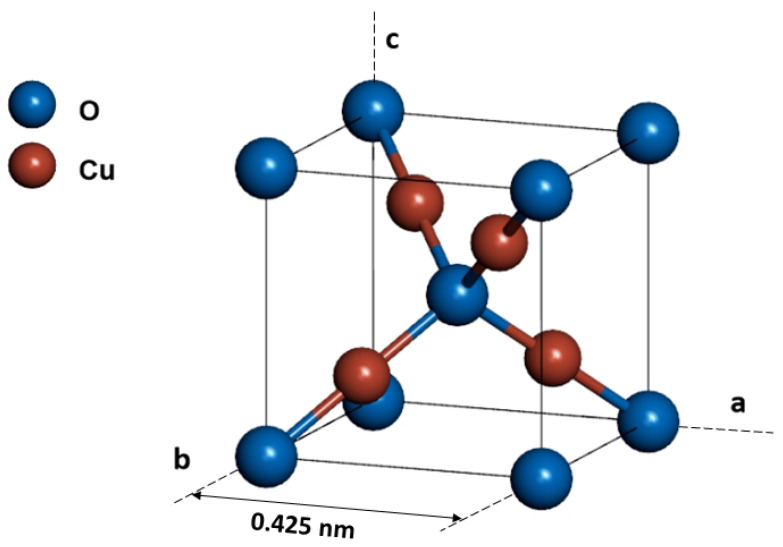
Unit cell of Cu_2_O, prepared based on JPCDS card # 01-074-1230.

Copper creates two stable oxides, cupric CuO oxide and cuprous Cu_2_O oxide [1]. Both are p-type semiconductors that show high absorption in the visible range of light, with an absorption coefficient value of order of 10^5^ cm^−1^ [2]. Cuprous Cu_2_O oxide has a direct forbidden energy band gap of 2.09 eV at room temperature [3]. It crystallizes in the Pn3m cubic system (space group no. 224) with a lattice constant of 0.425 nm, as presented in Figure 1. This material has attracted a lot of interest as it has potential to be used in sensors [4,5], photocatalysis and water splitting [6,7,8], and photovoltaics [9,10,11].

In particular, copper Cu_2_O oxide is attractive as a possible photovoltaic absorber in thin-film solar cells. Many researchers performed the preparation of solar cells with different methods using Cu_2_O in various forms. Cui and Gibson [12] prepared a nanopillar construction of a solar cell based on the ZnO/Cu_2_O heterojunction. ZnO is an n-type semiconductor that acts as an emitter layer (also called window layer), while Cu_2_O is a p-type semiconductor and is the absorber layer. The electrodeposition method was used to obtain both ZnO nanopillars and Cu_2_O polycrystalline thin films on top. Such a system with indium tin oxide (ITO) and Au as electrodes showed the efficiency of energy conversion equal to 0.88%. Although not the best performance, the described method is low-cost and scalable for the future production of Cu_2_O-based solar cells. Thin films of Cu_2_O for photovoltaic cells are also prepared with other methods, such as pulsed laser deposition [13,14], magnetron sputtering [15,16], and pyrolysis [17].

Systems with a different emitter—TiO_2_—were prepared by Wisz et al. [18] and Pavan et al. [17]. The first group used the reactive magnetron sputtering technique to firstly prepare TiO_2_ and then Cu_2_O thin films on ITO substrate. The heterojunctions did not show photovoltaic activity, leaving room for the improvement of either this method or the materials used. The second group used the spray pyrolysis method to deposit TiO_2_ and Cu_2_O thin films. In [17], the authors studied different thicknesses of the Cu_2_O absorber layer and found that a 300 nm layer is a good absorber for higher-energy photons (above 2.5 eV). The highest open circuit voltage and short-circuit current obtained were 350 mV and 0.4 mA/cm^2^, respectively.

Minami et al. [19] created a solar cell with a Cu_2_O absorber that had 6% efficiency, which for cells using copper oxides is a very good performance. The cuprous oxide thin films, obtained with the thermal oxidation of copper, were doped with Na. As the n-type layer, aluminum gallium oxide was used. A decent performance was achieved by the doping of material and for the successful adaptation of copper oxides in photovoltaics and other applications; this type of modification is crucial, as was shown by Zivkovic et al. [20]. The simple calculation of possible efficiency based on Shockley–Queisser theory [21] is not sufficient; the nature of electronic and optical properties of oxides must be taken into account, and type of transitions occurring in the materials plays an important role.

The abovementioned data indicate that the doping of Cu_2_O is a good solution to improve the properties of this oxide. Here, we test the method of ion implantation to modify the properties of Cu_2_O thin films with the incorporation of Cr ions. The ions were implanted with different dosages at energy of 10 keV. The thin-film samples were deposited using reactive DC magnetron sputtering. We used the X-ray diffraction method (XRD) to determine the structure of deposited and implanted films. The optical properties of the oxide were studied with spectrophotometry in the UV-VIS range and with spectroscopic ellipsometry. The ellipsometry method allowed us to model the thickness of the samples and map the uniformity of the surface of the samples. We simulated the interaction of implanted Cr ions with Cu_2_O oxide using Stopping and Range of Ions in Matter software [22]. The main goal of the study was to determine the influence of Cr impurity on the surface and subsurface morphology, structure, and optical characteristics.

## 2. Results and Discussion

### 2.1. XRD Study

**Figure 2:**
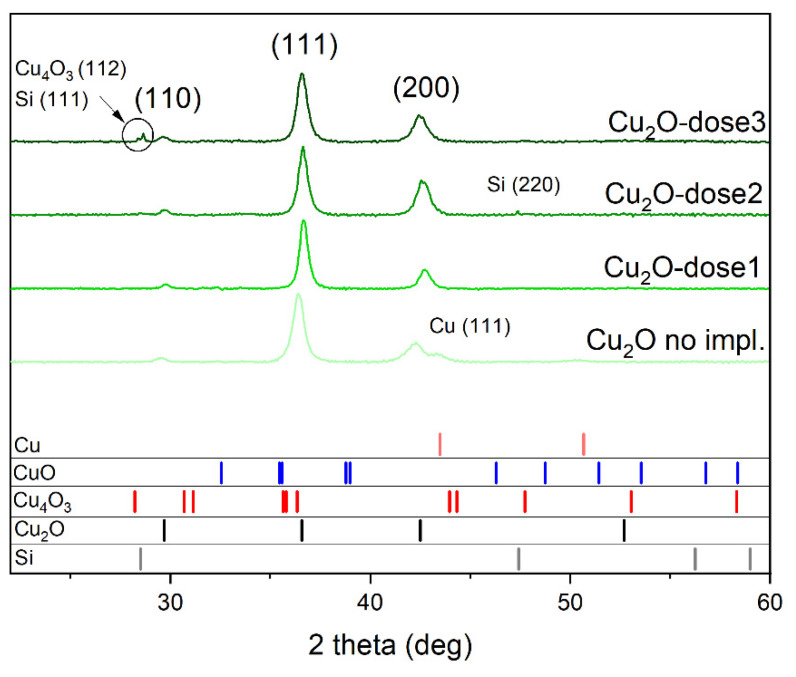
X-ray diffractograms of non-implanted and implanted Cu_2_O samples. In the lower part of the graph, reference patterns of copper, copper oxides, and substrate material are shown.

The diffractograms of four samples, the as-deposited reference and three implanted with different doses, are presented in Figure 2. All samples show three peaks corresponding to Cu_2_O (JPCDS card #01-074-1230): (110), (111), and (200). For the non-implanted sample, a peak from Cu is also seen; this might indicate that some additional copper was deposited during sputtering, not only the oxide, yet the Cu_2_O peaks are distinct and clearly visible. The sample implanted with the highest dose shows two small peaks around 28°, which come from the substrate (JPCDS card #00-003-0544) and another copper oxide—Cu_4_O_3_ (JPCDS card #00-0491830). This latter indicates a phase change induced by implantation and also can suggest some damage done to the layer by implanted ions so that the substrate signal is stronger. A small peak from Si (JPCDS card #00-003-0544) is also visible for film implanted with medium dose.

All visible Cu_2_O peaks were used to calculate the lattice constant *a*. This simple calculation was based on Bragg’s law and the relation between d-spacing *d*_hkl_ and the lattice constant *a* for cubic structure [23].

The calculated lattice constant *a* were 0.427 nm, 0.424 nm, 0.425 nm, and 0.426 nm for non-implanted, dose 1, dose 2, and dose 3 samples, respectively. These values of lattice constant are very close to the reference, which is 0.425 nm (JPCDS card #01-074-1230).

We calculated the microstrain of the structure and the size of crystallites fitting the (111) peak. The fitting function was pseudo-Voigt [23]:

(1) $$y=y_{0}+A\left(m_{u}\frac{2}{\pi }\frac{w_{L}}{4{\left(x-x_{c}\right)}^{2}+w_{L}^{2}}+\left(1-m_{u}\right)\left(\frac{\sqrt{4ln2}}{\sqrt{\pi }w_{G}}e^{-\frac{4ln2}{w_{G}^{2}}{\left(x-x_{c}\right)}^{2}}\right)\right)$$

where *x* is an angle of Bragg peak position, *x*_c_ is an angle of peak center position, *y*_0_ is an offset value, *A* is an area of the peak, *m*_u_ is the Lorentz fraction, *w*_L_ is the peak width of Lorentz function, and *w*_G_ is the peak width of Gauss function. The Gauss and Lorentz width of peak can be used to calculate microstrain *ε* and crystallite size *D*:

(2) $$\varepsilon =\frac{w_{G}}{4\tan\theta }$$

(3) $$D=\frac{\lambda }{w_{L}\cos\theta }$$

The results of the calculations are presented in Table 1.

The XRD results show that phase change could have been caused by the implantation. The work of Bind et al. [24] considers the phase transformation induced by N ion implantation. First, partly amorphous copper oxide was transformed to crystalline Cu_2_O due to nitrogen implantation, and higher-dose crystalline CuO was created which for the largest dose of ions transformed into a partly amorphous CuO phase. A similar mechanism might take place in the case of Cr implantation into Cu_2_O. This could explain the presence of Cu_4_O_3_ oxide, the properties of which are intermediate between CuO and Cu_2_O, as it contains Cu in both the Cu(I) and Cu(II) state. This oxide is also a metastable phase and is very difficult to obtain synthetically [25]. No clear dependence of microstrain or crystalline size on the dose of implantation was found (Table 1), although it appears that the implantation decreased the strain present in the oxide structure. It could be related to the presence of defects created during implantation and immediate strain relaxation.

### 2.2. SRIM Simulation

The SRIM calculations provide information about many features of the transport of ions in matter. The main aspects which can be recognized with this software package are the electrical and nuclear stopping powers of ions and the range and straggling distribution of ions in target. The electronic and nuclear stopping powers describe the interaction of incident ions with bound electrons and nuclei of the target atoms. The ion beam also causes damage to solid targets by atom displacement which can be simulated with the SRIM package. For calculations, as input parameters, we need the target density and composition. Every element has a different lattice, surface binding energy, and dissipation energy. This information combined with information about spatial distribution create input into the software [26].

**Figure 3:**
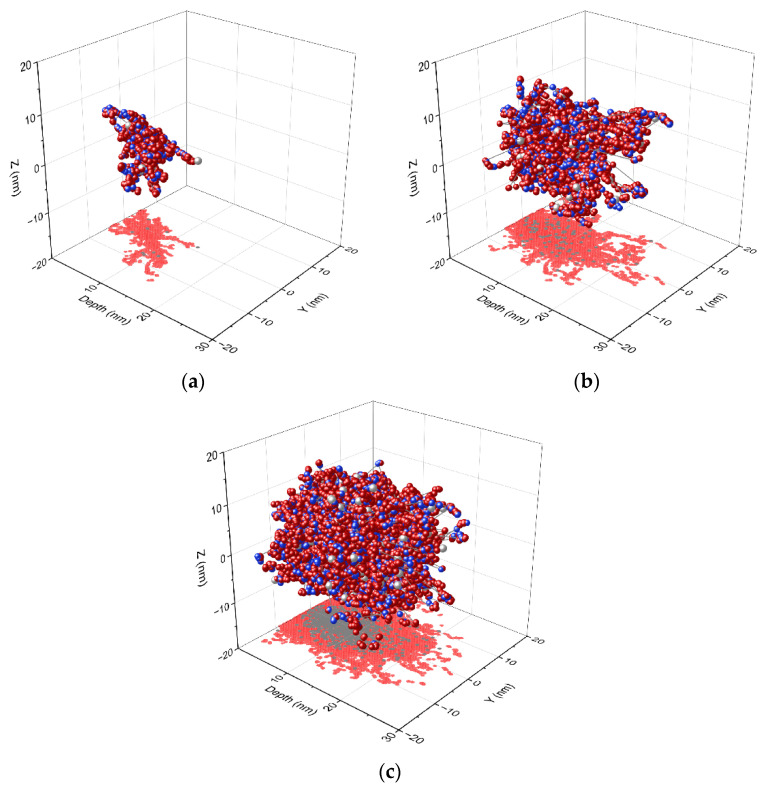
Ion and atom recoils for (**a**) 10, (**b**) 100, and (**c**) 1000 Cr ions. Blue balls are O atoms, red Cu atoms, and gray Cr ions; the projection: red are atoms O and Cu, and gray is Cr ions.

The influence of implantation was simulated using SRIM software. Here, results visualizing ion recoil collisions are presented. The simulation was performed for 30,000 random ions that all entered the material at the same spot. In Figure 3, the result of the implantation of 10, 100, and 1000 ions is presented. The calculated longitudinal projected range of ions is 7.3 ± 3.8 nm, which is the peak concentration depth. The Cr ions of 10 keV will reach 26 nm depth in Cu_2_O according to the simulation. In Figure 3, none of the Cr atoms reach depths more than the calculated 26 nm.

Each implanted ion hits an atom and gives energy to it which is dissipated as heat or is transferred to the next atoms. Some atoms can create numerous recoil cascades that result in disorder in the crystal lattice of an oxide. In Figure 3, the recoils for 10, 100, and 1000 ions are presented. The samples were implanted with at least 1 × 10^14^ ions (as the surface of the sample is approximately 1 cm^2^). It can be imagined how much energy and crystal lattice damage can introduce such an amount of ions, leading in critical cases even to the amorphization of a material. Unfortunately, it is not possible to describe these processes with the available software and computing power, which allowed us to perform the simulation of recoils for a maximum of 1000 ions. Calculations for a larger number of ions require more sophisticated tools and are computer-time-demanding.

### 2.3. Ellipsometry

The ellipsometry method is based on the analysis of polarized light. Two parameters are measured, *Psi* and *Delta*, which describe the polarization of a beam of light. Using mathematical formulas, it is possible to model the optical and physical properties of materials that influence the polarization of an analyzed beam. The most important relation that governs this method is the Kramers–Kroenig relation [27] between real *ε*_1_ and imaginary *ε*_2_ parts of dielectric function:

(4) $$\varepsilon_{1}\left(\omega \right)=1+\frac{2}{\pi }\;P\int_{0}^{\infty }\frac{\omega^{\prime }\varepsilon_{2}\left(\omega^{\prime }\right)}{{\omega^{\prime }}^{2}-\omega^{2}}d\omega^{\prime }$$

(5) $$\varepsilon_{2}\left(\omega \right)=-\frac{2\omega }{\pi }\;P\int_{0}^{\infty }\frac{\varepsilon_{1}\left(\omega^{\prime }\right)-1}{{\omega^{\prime }}^{2}-\omega^{2}}d\omega^{\prime }$$

where *P* is the principal value of the integral:

(6) $$P\int_{0}^{\infty }d\omega^{\prime }\equiv \underset{\delta \to 0}{\lim}\left(\int_{0}^{\omega -\delta }d\omega^{\prime }+\int_{\omega +\delta }^{\infty }d\omega^{\prime }\right)$$

*ω* is the angular frequency and *ω*’ is the complex angular frequency.

**Figure 4:**
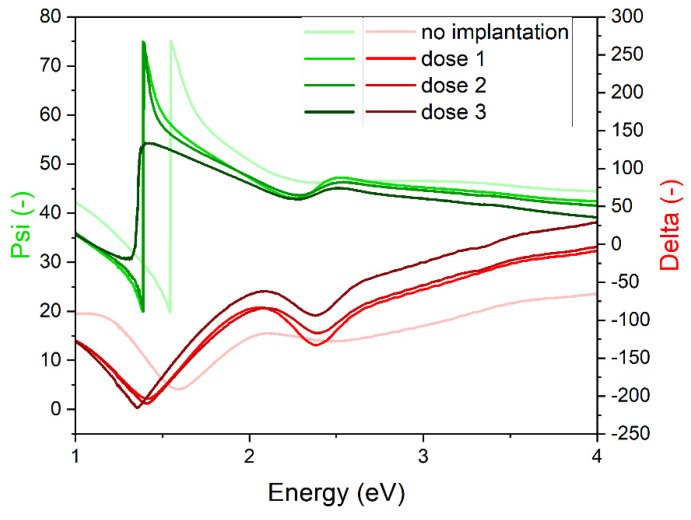
*Psi* and *Delta* measured for representative points of all Cu_2_O samples deposited on Si.

Measured data for representative points of tested samples are presented in Figure 4. The difference for Psi and *Delta* values was found between the non-implanted sample and implanted samples. The implanted samples’ *Delta* value has similar shape, while the non-implanted sample *Delta* shows less maxima. The *Psi* for dose 1 and dose 2 samples is almost identical; for dose 3, the shape is changed, and for non-implanted sample, the line is shifted towards higher energy.

In order to obtain information about the thickness and optical properties of the sample from ellipsometric raw data, one has to construct a model of the desired material. In the literature, authors used Lorentz [28] and Tauc–Lorentz [14] oscillators to model Cu_2_O. Here, we decided to use Tauc–Lorentz oscillators because in a preliminary fitting, it gave better results. The Tauc–Lorentz oscillator equation is [29]:

(7) $$\varepsilon_{T-L}\left(E\right)=\varepsilon_{1}-i\varepsilon_{2}\;\varepsilon_{2}=\left[\frac{Amp\;E_{0}\;Br{\left(E-E_{g}\right)}^{2}}{{\left(E^{2}-E_{0}^{2}\right)}^{2}+Br^{2}E^{2}}\cdot \frac{1}{E}\right],\;E>E_{g}\;\varepsilon_{2}=0,\;E\leq E_{g}\;\varepsilon_{1}=\frac{2}{\pi }P\int_{E_{g}}^{\infty }\frac{\xi^{\prime }\varepsilon_{2}\left(\xi \right)}{\xi^{2}-E^{2}}d\xi$$

**Figure 5:**
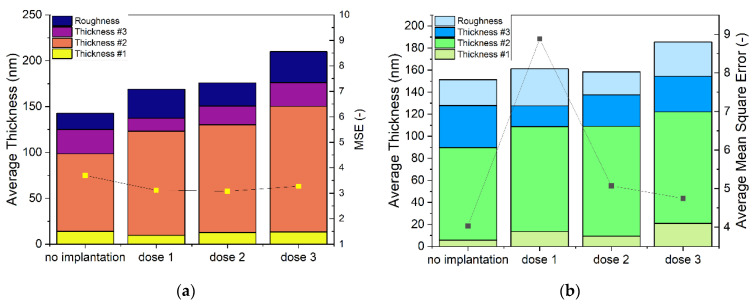
Thickness and fit figure of merit—mean squared error of fits performed for (**a**) representative points of maps, (**b**) whole maps.

After the first B-spline fitting of the non-implanted sample, a model for Cu_2_O was created based on four Tauc–Lorentz oscillators. The model used for samples was based on five layers: substrate—Si, the interface layer between substrate and film (layer #1), the body of the film (layer #2), the top layer (layer #3), and the roughness layer (which is 50% void and 50% top layer). The thickness of representative points and of whole maps’ fitting are presented in Figure 5.

**Figure 6:**
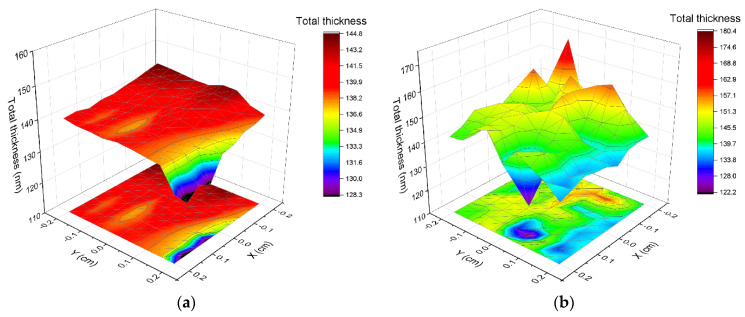

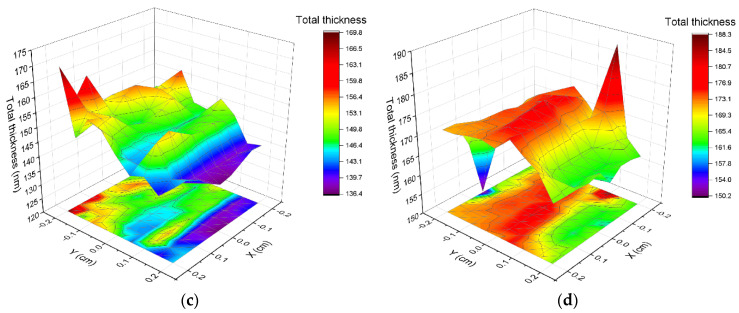
Total thickness parameter calculated for maps of Cu_2_O samples (**a**) non-implanted, (**b**) dose 1, (**c**) dose 2, and (**d**) dose 3.

The total thickness of sample is the sum of layer #1, layer #2, and layer #3 and half of the roughness layer. The total thickness results for maps of all four samples are presented in Figure 6. Each sample shows some points that are very different from most of the layer, e.g., for the non-implanted sample near X 0.2, and Y 0.2 corner. The map of the non-implanted sample (Figure 6a) shows uniform distribution over the whole area, except for some points in one edge. Implanted samples show less uniformity (Figure 6b–d), which indicates changes in optical properties induced by implantation. The mean squared error (MSE) value for map fits were fair, as they were <10, which for a map fitting is a good result.

**Figure 7:**
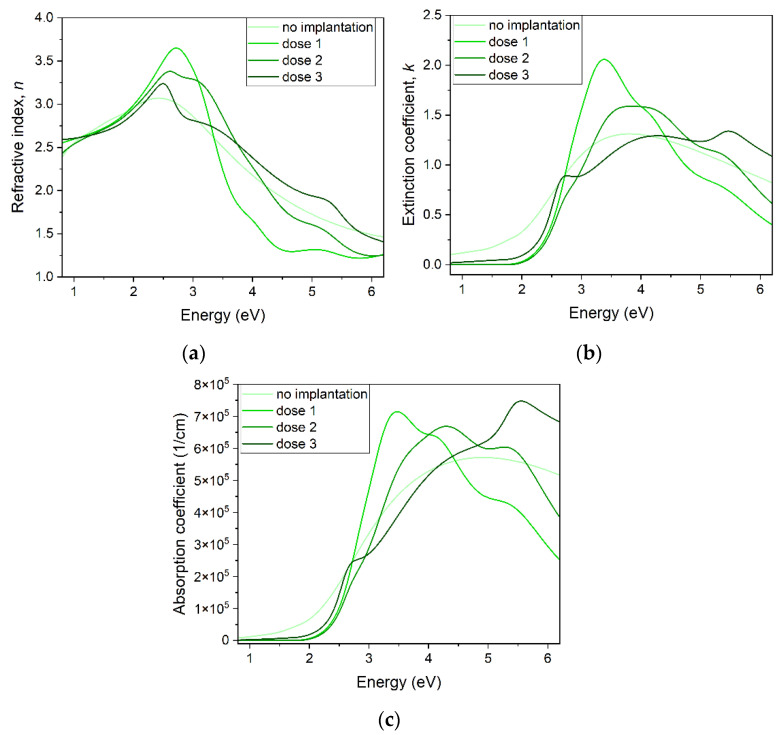
Optical properties of Cu_2_O samples calculated from ellipsometric model fitting: (**a**) refractive index *n*, (**b**) extinction coefficient *k*, (**c**) absorption coefficient α.

Representative points for all samples were modeled to determine the optical properties of oxide. The *n*, *k*, and absorption coefficient calculated from the fitted model are presented in Figure 7. The smoothest dependence of *n*, *k*, and absorption coefficient on energy is observed for the non-implanted sample. The implanted samples show additional maxima, the number and position of which depend on the implanted dose.

The ellipsometry results show that the total thickness of a modeled sample increases with the dose of ions. The deposited samples were all of the same thickness, as they were deposited in the same conditions and for the same time. In Figure 5a, it is seen that the thickness of the roughness layer and layer #3 is larger the higher the dose of implantation, while lower layers do not show this tendency. Introduced Cr increases the thickness of films, with the most influence on the layers nearest to the surface. The modeled roughness of the samples increases, which is in accordance with the AFM results.

The *n*, *k*, and absorption coefficient optical parameters show different energy dependence related to the implantation dose. The Tauc–Lorentz model consisted of four oscillators that had energy at around E_1_ = 2.5 eV, E_2_ = 3 eV, E_3_ = 4 eV, and E_4_ = 5.5 eV, although these energies differed between the implanted samples. It seems that with the increasing dose of implantation, the amplitude of E_2_ and E_3_ increases first, then the amplitude of E_2_ decreases and of E_4_ increases, and the amplitudes of E_1_ and E_4_ increase, and the others decrease. An oscillator can indicate some kind of transition that occurs at the indicated energy. Indeed, according to Ito et al. [30], four transitions could be observed at energies: 2.624 eV, 2.755 eV, 3.5 eV, and 4.3 eV. These values are smaller than ones fitted by us. An improvement to this optical property analysis would be to prepare more samples and perform ellipsometric measurement for more than one angle.

### 2.4. Absorbance

**Figure 8:**
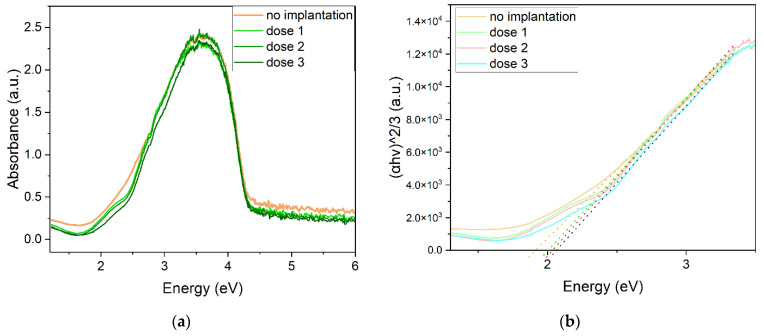
(**a**) Absorbance of Cu_2_O thin films, (**b**) Tauc plot for Cu_2_O.

The absorbance of the non-implanted sample differs from the absorbance of the implanted sample for an energy range below 2.5 eV and above 4 eV. In the spectral range, where the intensity of absorbance is the highest, the values for all samples are close. However, the dose 3 sample has quite a distinctively lower absorbance than the others (Figure 8a).

A Tauc plot was created for a direct forbidden transition (γ = 3/2). From this method, the calculated energy band gaps are: 1.91 eV, 2.00 eV, 2.04 eV, and 2.06 eV for non-implanted, dose 1, dose 2, and dose 3 samples, respectively. The optical energy band gap increases with the dose of implanted ions. For implanted samples, an envelope is seen around 2 eV, which may indicate another transition occurring in the material. In the case of the non-implanted sample, this effect is not observed (Figure 8b).

### 2.5. Infrared Spectroscopy

**Figure 9:**
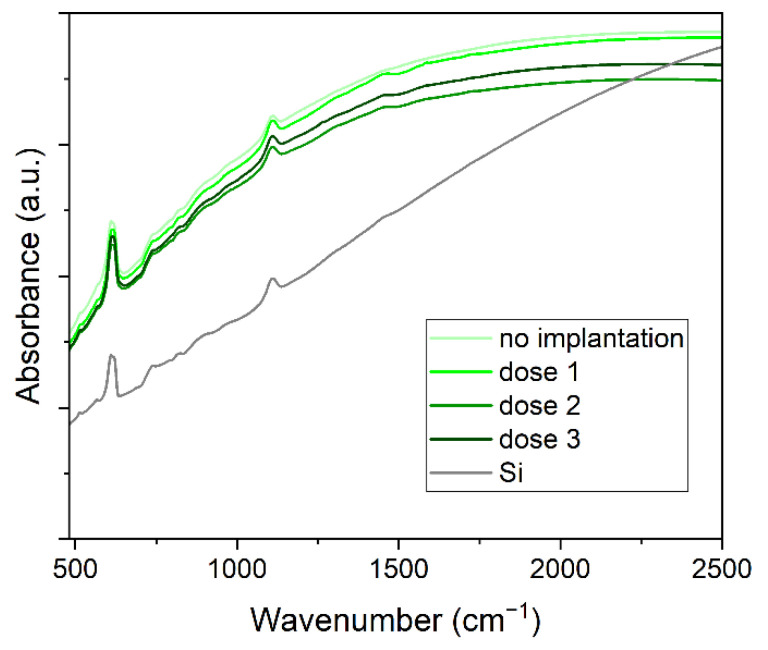
Absorbance of Cu_2_O samples and Si substrate measured with infrared spectroscopy.

The results of the Fourier transform infrared spectroscopy measurement are presented in Figure 9. The comparison of the signal from Cu_2_O samples (as deposited and implanted) with the reference spectrum of Si substrate demonstrates that the measured spectra are dominated by the signal from silicon. Cuprous oxide has one allowed vibration around 620 cm^−1^ [31], which is not seen in our samples. The absorbance of all Cu_2_O samples is larger than the silicon signal, as can be expected from a substrate with a film of highly absorbing oxide, which Cu_2_O is. Additionally, implanted samples show less absorption than the non-implanted sample in infrared spectroscopy.

### 2.6. Atomic Force Microscopy

**Figure 10:**
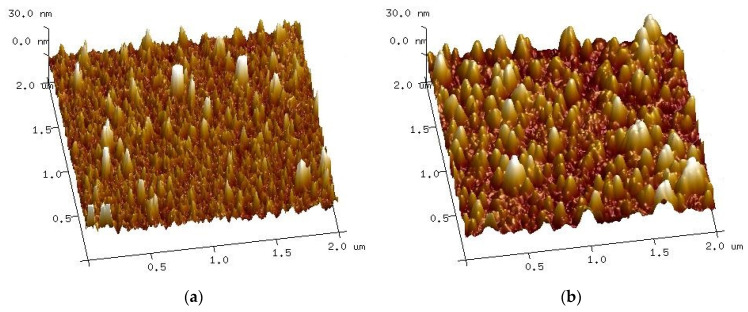

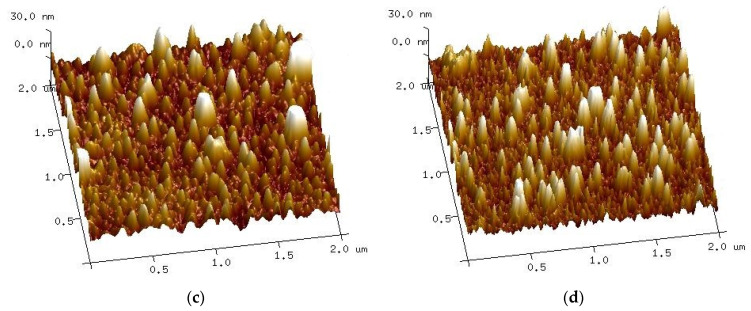
Atomic force microscopy 3D topographic maps for samples (**a**) non-implanted, (**b**) dose 1, (**c**) dose 2, and (**d**) dose 3.

In Figure 10, atomic force microscopy (AFM) measurements are presented. It can be seen that in case of implanted samples, some clusters or bigger crystallites emerge. The roughness measured with the AFM method is shown in Table 2. The roughness of the films was estimated as two parameters *R*_a_ (arithmetical mean deviation of the assessed profile) and *R*_max_ (maximum valley depth below the mean line). The surface analysis with AFM shows that the average roughness is higher for implanted than non-implanted samples.

## 3. Materials and Methods

Thin films of Cu_2_O were deposited using the reactive magnetron sputtering method. Two substrates were used: silicon and glass to perform different studies on deposited layers. Before deposition, the substrates were prepared by cleaning in warm water with soap, rinsing with isopropanol, and submerging in alcohol for 20 min in an ultrasonic bath, and finally drying with N_2_. As a target during deposition, a copper cathode (99.95% purity, Kurt J. Lesker, Hastings, UK) was used, and the power of discharge was 50 W. Before the deposition of thin films, a presputtering process was carried out in Ar and then Ar + O_2_. This process allowed us to clean the target and create ionized gas that accelerated the deposition. The pressure during deposition was at the level of 1.0 × 10^−2^ mbar, and the flow of gases was 18 sccm and 2 sccm for Ar and O_2_, respectively. Deposition time was around 3 min to achieve the thickness of 130 nm. The substrates were heated to 150 °C. The thickness was measured with a Taly-step profilometer of Rank Taylor Hobson to test the deposition processes and to allow the appropriate time of deposition to be chosen.

Ion implantation was performed at the Henryk Niewodniczański Institute of Nu-clear Physics Polish Academy of Sciences in Krakow. Hydrated chromium trichloride was used as the source of Cr ions. The implantation energy was 10 keV, and the dose of ions was: 1 × 10^14^ (dose 1), 5 × 10^14^ (dose 2), 1 × 10^15^ (dose 3) ion/cm^2^.

The structural properties and phase composition of films were determined with X-ray diffraction using a PANalytical X’Pert PRO diffractometer (Malvern Panalytical, Malvern, UK) with a Cu anode (0.154 nm radiation wavelength). The measurement step was 0.05° with a time per step of 8000 s.

FTIR measurements were performed in transmission mode; 256 scans with 4 cm^−1^ resolution in the range of 950 to 400 cm^−1^ were performed. The surface topography of thin films was investigated using atomic force microscopy (AFM, Dimension Icon Bruker, Santa Barbara, CA, USA) working in the tapping mode.

Measurements of spectroscopic ellipsometry were performed with a J.A. Woollam M 2000 ellipsometer (J.A. Woolam, Lincoln, NE, USA) at angle of 70°. A map was measured for all samples: the maps were 5 × 5 mm with the step of 0.5 mm and 0.5 mm of margin—81 points were measured. Spectrophotometry measurements of absorbance were performed with an AvaLight-DH-S-BAL source and an AvaSpec-ULS-RS-TEC detector (Avantes, Apeldoorn, The Netherlands) in transmission mode.

SRIM calculations were made for the 100 nm layer of Cu_2_O. The parameters used in the simulation were: density of film 6.00 g/cm^3^, atomic ratio of Cu and O atoms 2:1, energy of Cr ions 10 keV, and incident angle of ion beam 0°. The simulations were calculated for 30,000 ions.

## 4. Conclusions

We deposited thin films of cuprous oxide with no other phases, as the XRD study proved. The implantation process decreased the microstrain of the crystal lattice but had no clear influence on the crystallite size. Phase change was observed caused by ion implantation as peaks of Cu_4_O_3_ emerged.

The absorption of implanted films is lower for both infrared and UV-VIS ranges of light. The optical band gap of material increases from 1.91 eV to 2.06 eV. Using different doses of ions, we can control the band gap of the oxide. Regarding the optical properties, refractive index, and extinction coefficient of oxide change with implantation, the ellipsometric model consisting of four oscillators shows different characteristics for each sample. The calculated absorption coefficient values are in the order of 10^5^ cm^−1^. The Cr ion implantation reduces the absorption of cuprous oxide, which is not favorable for photovoltaic application. However, annealing and restoring the crystal structure of the oxide may show a different result, and this could be a next step to study.

The implantation process increases the roughness of deposited oxide. AFM measurement showed that the mean roughness increases from 3.49 nm for the non-implanted sample to about 5 nm for implanted samples. Spectroscopic ellipsometry model fitting showed that the thickness of the two top layers in the model increases with the dose of ions, indicating higher roughness and stronger influence on subsurface-region properties.

For future experiments, it would be useful to measure the electrical properties of modified Cu_2_O thin films, e.g., with Hall effect measurements. Performing surface-sensitive methods such as secondary ion mass spectrometry or X-ray photoelectron spectroscopy could provide Cr detection, and this is planned as future studies. More experiments with higher dosages and energy of implanted ions could give more information about the influence of Cr on Cu_2_O properties, because with the dosage used in this work, no Cr or its compounds were identified in the material. Moreover, a creation of simple heterostructures with other oxides (ZnO or TiO_2_) could give additional information on this type of material modification and determine whether it is profitable for application in photovoltaics or photocatalysis.

## Figures and Tables

**Table 1:** The results of calculation for (111) using fitting to pseudo-Voigt function.

Sample	Microstrain (-)	Crystallite Size (nm)
no impl.	0.0093	15
dose 1	0.0065	19
dose 2	0.0083	18
dose 3	0.0075	14

**Table 2:** Roughness of Cu_2_O thin films measured with atomic force microscopy.

Sample	*R*_a_ (nm)	*R*_max_
non impl.	3.49	62.7
dose 1	5.18	51.2
dose 2	5.32	79.9
dose 3	5.08	58.5

## Data Availability

Data supporting this article is available upon request from corresponding author.
